# Contrasting effects of climate change on denitrification and nitrogen load reduction in the Po River (Northern Italy)

**DOI:** 10.1007/s11356-024-34171-3

**Published:** 2024-07-18

**Authors:** Maria Pia Gervasio, Elisa Soana, Anna Gavioli, Fabio Vincenzi, Giuseppe Castaldelli

**Affiliations:** Department of Environmental and Prevention Sciences, University of Ferrara, Via Luigi Borsari 46, 44121 Ferrara, Italy

**Keywords:** Climate change, Water temperature, Nitrogen, Denitrification, DNRA, Po River

## Abstract

An increase in water temperature is one of the main factors that can potentially modify biogeochemical dynamics in lowland rivers, such as the removal and recycling of nitrogen (N). This effect of climate change on N processing deserves attention, as it may have unexpected impacts on eutrophication in the coastal zones. Intact sediment cores were collected seasonally at the closing section of the Po River, the largest Italian river and one of the main N inputs to the Mediterranean Sea. Benthic oxygen fluxes, denitrification, and dissimilatory nitrate reduction to ammonium (DNRA) rates were measured using laboratory dark incubations. Different temperature treatments were set up for each season based on historical data and future predictions. Higher water temperatures enhanced sediment oxygen demand and the extent of hypoxic conditions in the benthic compartment, favoring anaerobic metabolism. Indeed, warming water temperature stimulated nitrate (NO_3_^−^) reduction processes, although NO_3_^−^ and organic matter availability were found to be the main controlling factors shaping the rates between seasons. Denitrification was the main process responsible for NO_3_^−^ removal, mainly supported by NO_3_^−^ diffusion from the water column into the sediments, and much more important than N recycling via DNRA. The predicted increase in the water temperature of the Po River due to climate change may exert an unexpected negative feedback on eutrophication by strongly controlling denitrification and contributing to partial buffering of N export in the lagoons and coastal areas, especially in spring.

**Graphical Abstract:**

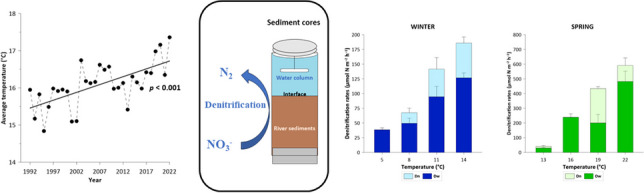

## Introduction

Rivers are heavily affected by anthropogenic activities, particularly by nitrogen (N) input from agricultural sources, mostly in the form of nitrate ion (NO_3_^−^) (Lassaletta et al. 2009; Sutton et al. 2011). Nitrogen loads can affect the trophic state of rivers and, when delivered to terminal water bodies, fuel eutrophication and trigger algal blooms in both transitional and coastal zones (Dodds 2006; Glibert 2017). The generation of N loads, their transport across land-river and river-sea interfaces, and eutrophication dynamics are strongly influenced by land use, river morphology, and hydrological and thermal conditions, all of which are subject to climate change (Hou et al. 2019; Romero et al. 2013; Tu 2009).

Extreme hydrological events such as floods and droughts, which have increased in frequency in recent years, directly impact river discharge and the generation and delivery of nutrient loads to water bodies, transport dynamics within the hydrological network, and microbial processes of N transformation and removal (Abily et al. 2021; Zheng et al. 2023). River sediments are active sites for biogeochemical reactions (Triska and Higler 2009), such as denitrification and DNRA. The denitrification is the anaerobic respiration that converts NO_3_^−^ to di-nitrogen gas (N_2_), the final product, and nitrite (NO_2_^−^) and nitrous oxide (N_2_O), as intermediate products. It is the most important process supporting the self-depuration capacity of rivers, removing NO_3_^−^ permanently from the ecosystem (Hill 2023; Piña-Ochoa and Álvarez-Cobelas 2006; Seitzinger 1988). In contrast, the dissimilatory nitrate reduction to ammonium (DNRA) is an alternative microbial pathway for NO_3_^−^ reduction, using the same substrates as denitrification (NO_3_^−^ and organic carbon), but recycling N in the ecosystem through the conversion of NO_3_^−^ to ammonium (NH_4_^+^) (Giblin et al. 2013).

River denitrification is controlled by several factors, such as oxygen concentration at the water-sediment interface, and the availability of NO_3_^−^ and labile organic carbon (Ballard et al. 2019; Hu et al. 2023; Piña-Ochoa and Álvarez-Cobelas 2006). One of the most important environmental drivers influencing denitrification is water temperature (de Klein et al. 2017; Veraart et al. 2011). Warming boosts denitrification alongside enzymatic reactions, but water temperature increase also has an impact on the process by regulating two of the aforementioned determinants of denitrification, i.e., oxygen concentration and availability of labile organic matter (de Klein et al. 2017; Speir et al. 2023). Indeed, all biogeochemical NO_3_^−^ dissimilatory pathways are affected by water warming, both as a direct effect of temperature on enzyme activity and an indirect effect on sediment redox conditions (Brin et al. 2017). Furthermore, the availability of labile organic carbon, oxygen, and redox conditions in sediments determine the relative roles of denitrification and DNRA in NO_3_^−^ removal or recycling in aquatic ecosystems (Nizzoli et al. 2010; Aalto et al. 2021).

Under climate change scenarios, a reduction in rainfall, runoff, and river flow is likely to lower NO_3_^−^ concentrations (Oduor et al. 2023), thereby limiting river denitrification. On the other hand, the expected increase in water temperature is likely to stimulate the process. Several studies have isolated the effect of temperature on denitrification through manipulative experiments (e.g., Silvennoinen et al. 2008; Velthuis and Veraart 2022; Speir et al. 2023). Nevertheless, there is a lack of systematic research predicting the consequences of warming on the self-depuration capacity of large lowland rivers, given their crucial role in processing anthropogenic N inputs along the land-sea continuum.

The Po is the largest Italian river in terms of watershed area and annual discharge at the closing section and is one of the major rivers in the Mediterranean region (Struglia et al. 2004). Its catchment is one of the most industrialized and intensively farmed catchments in the world (Moatti and Thiébault 2016), making it a hotspot for NO_3_^−^ pollution. The Po River contributes two-thirds of the total freshwater discharge and nutrient inputs conveyed to the Adriatic Sea (Grilli et al. 2020; Viaroli et al. 2018) and is the major basin for riverine N export to the Mediterranean Sea (Romero et al. 2021). In the last decades, the Po River basin was strongly affected by the increasing frequency of extreme events due to climate change (Appiotti et al. 2014; Coppola et al. 2014; Marchina et al. 2017). For example, in 2022, rainfall and river discharge were the lowest in historical records since 1961 (Montanari et al. 2023) and water temperature was the highest measured in the last two decades (Gervasio et al. 2023). Following these extreme hydrological and thermal conditions, experimental work was carried out on intact sediment cores sampled from the lower course of the Po River to measure benthic denitrification and DNRA rates. The aim of the present study was to assess the seasonal effect of temperature and NO_3_^−^ availability on the river buffering capacity against N pollution and eutrophication in the coastal zone.

## Materials and methods

### Study area

**Fig. 1:**
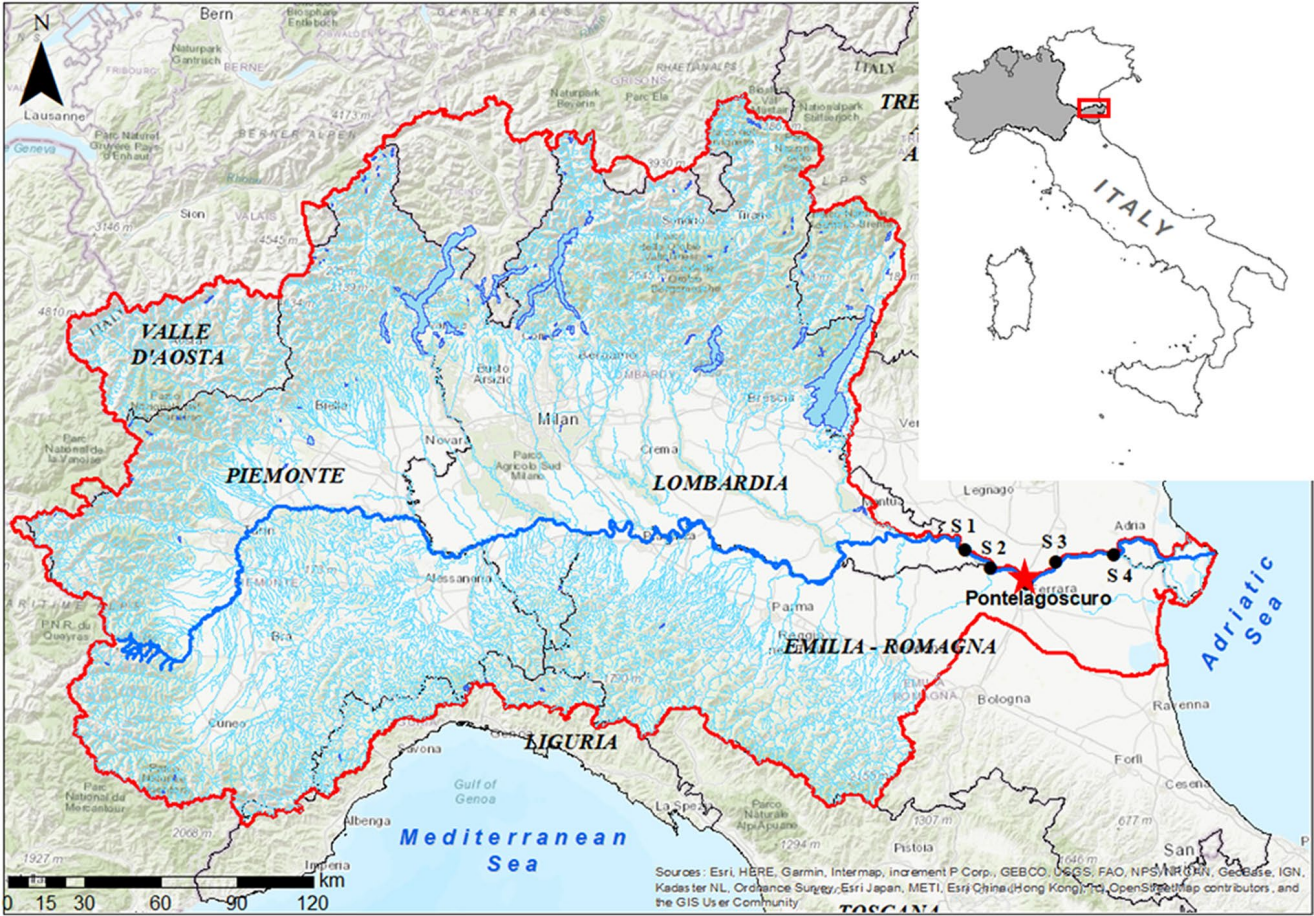
Map of the Po River course (blue line) and its basin (bordered with the red line and grey area on top) located in Northern Italy (modified by ArcGIS 10.8.2, ESRI). The red star indicates the sampling station in Pontelagoscuro. The black dots indicate the monitoring temperature stations belonging to the Regional Agency for Environmental Prevention of Emilia – Romagna, Lombardy, and Veneto Regions: Sermide (S 1), Stellata - Bondeno (S 2), Polesella (S 3), and Serravalle (S 4), situated in the province of Ferrara (Italy)

The Po River is the longest and most important Italian river, flowing from the Alps to the Adriatic Sea, with an average annual discharge of 1500 m^3^ s^−1^ at the Pontelagoscuro (Ferrara, 44°53′16.9″N, 11°36′26.6″E) measuring station, the official basin closing section (Fig. 1; Zanchettin et al. 2008). The basin covers an area of approximately 71,000 km^2^ across Italy, a quarter of the national territory. The river has more than 140 tributaries and a capillary network of artificial irrigation and drainage canals (Soana et al. 2019). The basin is subject to a mix of subcontinental and warm-temperature climates (the Mediterranean climate), which split the annual hydrological regime into two low-flow periods (winter and summer) and two recharging periods (spring and autumn) fed by snowmelt and rainfall (Coppola et al. 2014; Montanari 2012; Ravazzani et al. 2015). In recent decades, the Po River basin has experienced the effects of climate change, with an increase in extreme storm events (Brunetti et al. 2004; Domeneghetti et al. 2015; Giambastiani et al. 2017), long drought periods, and water temperature warming (Gervasio et al. 2022; Bonaldo et al. 2023; Soana et al. 2023). The Po River crosses the Po Valley, the most fertile and extensively cultivated area in Italy, and is the main source of irrigation water for crops. Since the 1960s, the intensification of agriculture and livestock farming has made the Po River the main source of nutrient inputs to the North Adriatic Sea, triggering algal blooms during warm periods (Penna et al. 2004; Spillman et al. 2007).

### Sampling activities

Sediment cores were sampled in the Po River at Pontelagoscuro (red star in Fig. 1), about 90 km from the main mouth to the Adriatic Sea, in winter (February), spring (May), summer (July), and autumn (November). Water column parameters (temperature, electrical conductivity, and oxygen concentration) were measured *in situ* using a multiparametric probe (YSI Model 85-Handheld Dissolved Oxygen, Conductivity, Salinity and Temperature System, Yellow Springs, OH, USA) during the sampling days. Sampling, pre-incubation, and incubation were performed according to standardized protocols (Dalsgaard 2000). The experimental design consisted of 25 intact sediment cores (plexiglass liners, internal diameter 4.5 cm, length 20 cm) collected for each seasonal sampling with a hand corner from the boat. The cores were sampled at depths ranging from 1.5 to 4 m, according to the different seasonal hydrological conditions of the Po River. All cores were leveled in order to have a sediment height of about 9 cm and an overlying water column of about 8 cm. After collection, intact sediment cores were submerged in tanks with site water continuously aerated using aquarium pumps and transported to the laboratory. Approximately 60 L of bottom water was collected and brought to the laboratory for core maintenance, pre-incubation, and incubation procedure. Five of the intact sediment cores were randomly assigned to each of the four temperature treatments and incubated in the dark to determine oxygen fluxes, denitrification, and DNRA rates. The remaining five cores were used for sediment characterization.

### Seasonal gradients of water temperature

**Fig. 2:**
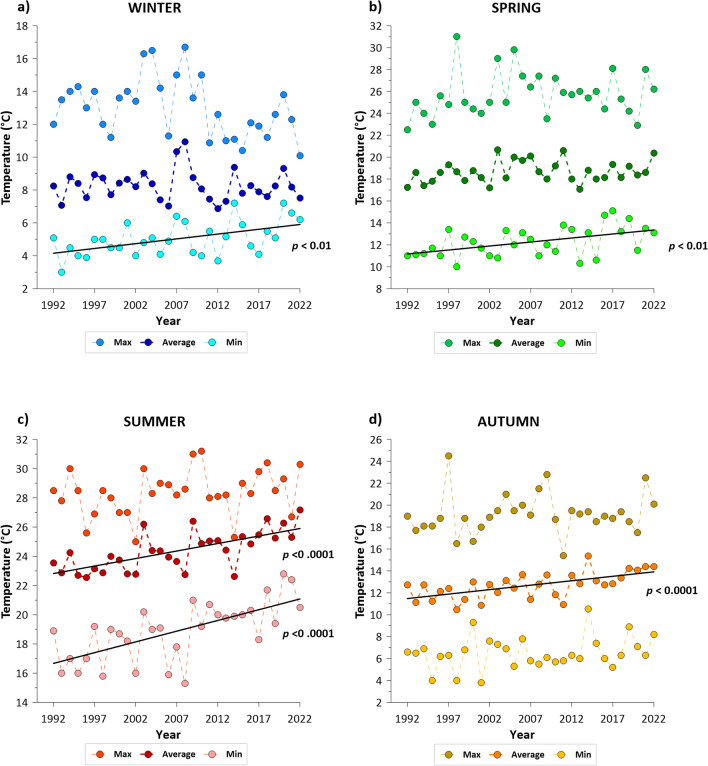
Historical average, minimum, and maximum water temperatures (°C) of the Lower Po River since the 1990s in each season, monitoring from Sermide to Serravalle sections. **a** Winter: January – March period. **b** Spring: April – June period. **c** Summer: July – September period. **d** Autumn: October – December. Solid lines show statistically significant trends

During each seasonal incubation, the temperature range was established from the historical temperature data of the Po River (1992–2022), monitored monthly by the Regional Environmental Protection Agencies of the Emilia – Romagna, Lombardy, and Veneto Regions (named ARPAE, ARPA of the Lombardy Region, and ARPAV, respectively) in five sections along a lowland 80-km stretch right upstream of the deltaic system: Sermide, (S1), Stellata–Bondeno (S2), Pontelagoscuro, our sediment sampling site, Polesella (S3) and Serravalle (S4) (black dots in Fig. 1). The Po River water temperature has gradual increased over the last decades (1992–2022; Fig. 2) and the upward trend was particularly marked in summer and autumn (nearly +5 °C; Fig. 2). Summer average temperature ranged from 22 (1996) to 27 °C (2022), showing a significant upward trend equivalent to a raise of 0.15 °C yr^-1^. In autumn, the trend was similar in slope to summer (0.16 °C yr-1), with values varying between 10 (1998) and 15 °C (2014). Although the average winter and spring temperature trends were not statistically significant, a slight increase was observed, with values ranging from 7 to 10 °C and from 17 to 20 °C, respectively. Extreme temperatures, both minimum and maximum, have also increased since the 1990s with a few exceptions. The minimum winter temperatures ranged from 3 to 7 °C, while spring values varied between 10 and 15 °C. Finally, summer minimum temperatures ranged from 15 to 23 °C, while the autumn minimum temperatures did not increase significantly. Maximum temperatures, on the other hand, did not show a significant trend, but there were some years with maximum temperatures higher than usual, such as 1998 for spring and autumn and the early 2000s for all seasons.

**Fig. 3:**
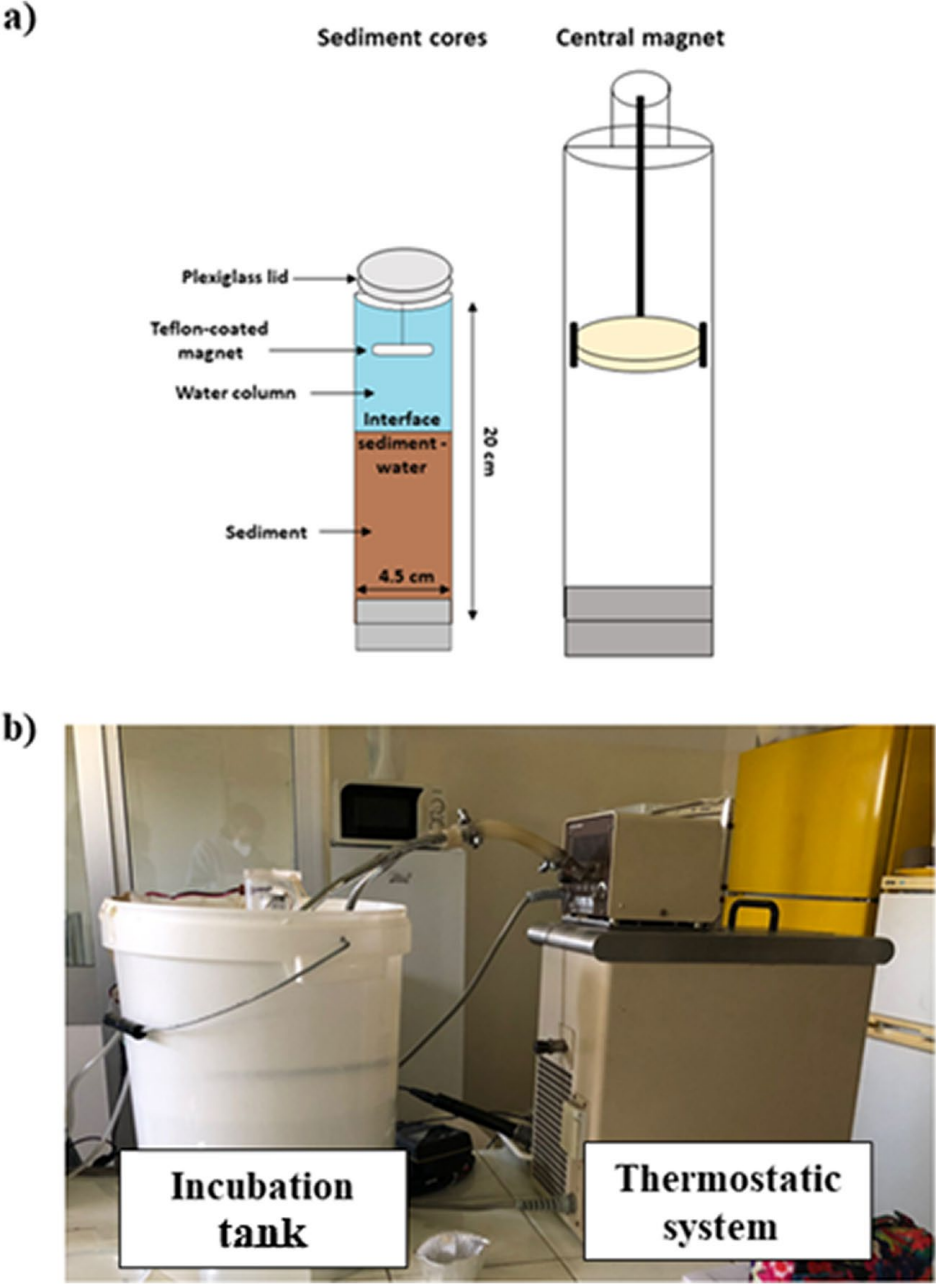
**a** Schematic representation of the incubation system with a central magnet and the example of sediment core situated in each tank. **b** Image of the incubation tank (on the left) connected to the thermostatic system (on the right) to regulate the experimental temperature for each seasonal incubation

Based on historical water temperatures time series, four different temperatures were applied in each seasonal incubation, covering the following ranges: 5–14 °C in winter (January, February, March), 13–22 °C in spring (April, May, June), 21–30 °C in summer (July, August, September), and 9–18 °C in autumn (October, November, December) (Table 1). The lower extreme of each seasonal range corresponded to the average of the seasonal minimum temperature measured in the Po River from the 1990s to the present, while the upper extreme of the range was established based on the maximum values expected in the near future owing to climate warming. In the period 2041–2070, air temperatures are expected to increase throughout the Po basin in all seasons, with positive anomalies of up to 3 °C (Vezzoli et al. 2015). Intermediate temperatures correspond to the most frequent seasonal mean temperatures over the last 30 years. The experimental temperature in the incubation tanks was controlled using a thermostat (Fig. 3b) and continuously monitored during each incubation using a multi-parameter probe.

**Table 1:** Water temperature treatments applied to sediment core incubations in each season (°C)

Season	T (°C)	T (°C)	T (°C)	T (°C)
*Winter*	5	8	11	14
*Spring*	13	16	19	22
*Summer*	21	24	27	30
*Autumn*	9	12	15	18

### Seasonal incubation procedure

Each core was equipped with a rotating Teflon-coated magnet driven by an external magnet connected to a motor (40 rpm). Inside the cores, the magnet was suspended a few centimeters above the sediment-water interface to gently mix the water column while avoiding resuspension (Fig. 3a). To allow for acclimatization, each target temperature level was set in the early afternoon of the day before the start of incubation. According to standardized protocols (Dalsgaard 2000; Owens and Cornwell 2016), intact sediment cores were incubated in batch mode to measure benthic dark oxygen fluxes (sediment oxygen demand, SOD). Dark incubations were chosen to simulate *in situ* conditions, where light penetration is limited by turbidity and the benthic compartment is in the dark (Braga et al. 2017). The water in each tank was replaced to maintain *in situ* dissolved nutrient concentrations. Before the incubation of the benthic fluxes, O_2_ was measured using a multiparametric probe inside each core. The water level in the tanks was lowered to a few centimeters below the top of the cores, and each liner was sealed with a plexiglass lid (Fig. 3a). Incubation times ranged from 1.5 (summer experiment) to 4 (winter experiment) hours and were set, based on preliminary tests, as the minimum time needed to detect significant changes in solute concentrations and to keep oxygen concentrations at the end of the incubation within 20% of the initial value (Dalsgaard 2000). In particular, the winter and autumn incubations lasted 4 h due to the lower experimental temperatures and lower microbial process rates, while the spring incubation lasted 4 h for the first temperature level (12 °C) and 3 h for the other three experimental temperatures. Finally, summer incubations lasted 2.5 h for the first temperature level (21 °C) and 1.5 h for the other temperatures.

At the end of the incubation period, the O_2_ concentration in each core was measured in the same manner as that at the beginning. After the first incubation, the water in the tanks was replaced and the cores were submerged for approximately 2 h to stabilize the system. In the second incubation, the isotope pairing technique (IPT; Nielsen 1992) was applied to measure the denitrification and DNRA rates. As in the first incubation, the water level in the tank was lowered to just below the top of the cores to isolate them. An aliquot (0.6 – 1.5 mL according to the water volume in each core and the ambient seasonal NO_3_^−^ availability) of a stock solution of 15 mM ^15^NO_3_^−^ (Na^15^NO_3_, Sigma Aldrich, ≥98 atom% enrichment) was added to each core to obtain a final ^15^NO_3_^−^ enrichment of 30 – 60%. Then, the cores were capped to start the IPT incubation. The NO_3_^−^ concentrations were measured in each core before and after the addition of ^15^NO_3_^−^ to calculate the ^14^N:^15^N ratio in the NO_3_^−^ pool. The incubation times were the same as those used for the flux incubations and were set to ensure that oxygen consumption was less than 20% of the initial concentration, which is a prerequisite for the IPT method application (Nielsen 1992).

At the end of the incubation period, the entire sediment column was mixed with the water column to homogenize the dissolved N_2_ pools in the water column and pore water. Slurry samples were transferred to glass-tight vials (12 mL, Exetainer®, Labco Limited, UK), flushing at least 3 times the vial volume, and fixed with 200 μL of 7 M ZnCl_2_ to stop the microbial activity. The IPT samples were analyzed for ^29^N_2_ and ^30^N_2_ using membrane inlet mass spectrometry (MIMS) (Bay Instruments, MD, USA; Kana et al. 1994).

An additional aliquot (30 mL) of the slurry from each core was used to determine the DNRA rate from the production of ^15^ NH_4_^+^ (Gervasio et al. 2023; Magri et al. 2022). To determine the exchangeable NH_4_^+^ pool, the slurry was treated with 2 g of KCl (2 M), shaken for 30 min, and centrifuged (1800 rpm for 15 min). The supernatant was then filtered through Whatman GF/F glass fiber filters, stored in 20 mL scintillation vials, and frozen for subsequent analysis. Slurry samples were purged with air to eliminate ^29^N_2_ and ^30^N_2_ pools produced during IPT incubation, transferred to 12 mL-Exetainers (Labco Limited, UK), and treated with an alkaline hypobromite solution to oxidize NH_4_^+^ to N_2_ (Warembourg 1993). After the oxidation procedure, the ^29^N_2_ and ^30^N_2_ concentrations were measured using MIMS.

### Calculation of SOD, denitrification, and DNRA rates

Hourly dark fluxes of O_2_ (SOD, μmol O_2_ m^−2^ h^−1^) were calculated from the rate of change of concentrations with time according to the following equation (Owens and Cornwell 2016):

1 $$\mathrm{SOD}=\frac{\left({\mathrm C}_0-{\mathrm C}_{\mathrm f}\right)\cdot V}{A\mathit\cdot t}$$

where *C*_0_ and *C*_f_ (μM) are the O_2_ concentrations at the beginning and end of incubation, respectively, A (m^2^) is the area of the sediment core, *V* (L) is the water volume of the sediment core, and *t* (h) is the incubation time, which was different for each temperature in each seasonal experiment.

Denitrification rates (μmol N m^−2^ h^−1^) were calculated on ^29^N_2_ and ^30^N_2_ production, as the equations below (Nielsen 1992):

2 $${D}_{15}=p29+2p30$$

3 $${D}_{14}={D}_{15}\cdot \left(\frac{p29}{2p30}\right)$$

where D_15_ is the denitrification rate of the added ^15^NO_3_^−^, D_14_ is the total denitrification rate of ^14^NO_3_^−^, and p29 and p30 are the production rates of ^29^N_2_ and ^30^N_2_, respectively. The total denitrification rate (Dtot) was divided into two components as follows:

4 $$D_{\mathrm{tot}}\;=\;D_{\mathrm w}\;+\;D_{\mathrm n}$$

5 $$D_{\mathrm w}\;=\;\left(\frac{{}^{14}NO_3^-}{{}^{15}NO_3^-}\right)\cdot D_{15}$$

6 $$D_{\mathrm n}=D_{14}-D_{\mathrm w}$$

where Dw (μmol N m^−2^ h^−1^) is the denitrification rate of NO_3_^−^ diffusing from the water column to the anoxic sediment layer, while Dn (coupled nitrification-denitrification; μmol N m^−2^ h^−1^) is the denitrification rate of NO_3_^−^ produced within the oxic sediment layer by nitrification.

Anammox (anaerobic ammonium oxidation) may interfere with the IPT calculations, leading to an overestimation of the rates, as the N_2_ produced by anammox cannot be distinguished from that produced by denitrification. Therefore, the independence of the measured genuine ^28^N_2_ production from the ^15^NO_3_^−^ concentration added to each core was checked to validate the IPT assumptions and to exclude a significant overestimation due to anammox (Risgaard-Petersen et al. 2003). In addition, several studies report that anammox usually accounts for only a small fraction of total N_2_ production in eutrophic freshwater ecosystems (Koop-Jakobsen and Giblin 2009; Racchetti et al. 2011; Trimmer et al. 2003; Wei and Zhang 2023).

DNRA rates were calculated according to Risgaard-Petersen and Rysgaard (1995), as follows:

7 $$\mathrm{DNRA}\;=\;\mathrm{pNH}_4^+\cdot\frac{D_{14}}{D_{15}}$$

8 $${\mathrm{DNRA}}_{\mathrm w}=\frac{{}^{14}NO_3^-}{{}^{15}NO_3^-}\cdot\mathrm{pNH}_4^+$$

9 $${\mathrm{DNRA}}_{\mathrm n}=\mathrm{DNRA}-{\mathrm{DNRA}}_{\mathrm w}$$

where pNH_4_^+^ is the production of ^15^NH_4_^+^, DNRAw represents the DNRA of NO_3_^−^ from the water column, and DNRAn is the DNRA rate coupled to nitrification.

### Sediment characterization

The cores for sediment characterization were extracted and sliced into two layers: upper 0–1 cm and 1–2 cm sections. Aliquots from the two layers were rapidly homogenized and subsamples of 5 mL were collected using plastic syringes to determine physical properties (density, volumetric water content, and porosity). Fresh sediments were dried at 50 °C to constant weight for 72 h and then they were set at 350 °C for 3 h into a muffle furnace. The dried samples were used to determine the organic matter content (OM, %) via weight loss during ignition.

### Statistical analyses

The correlation between water temperature and benthic fluxes of oxygen was analyzed using a parametric test (Pearson's correlation). The test was performed using the software SigmaPlot 15.0 (Systat Software, Inc., San Jose, CA, USA) and the overall significance level was set at *p* ≤ 0.05. Benthic fluxes of oxygen, denitrification, and DNRA rates were statistically analyzed using linear mixed effects (LME) to analyze differences in temperature and in seasons. The sample size was equal in all tests. The statistical analyses were run in RStudio (RStudio-2023.06.0-421), using the *lme4* package (Douglas Bates et al. 2015). The focus of the test was to compare the differences in seasonal SOD, denitrification, and DNRA rates by taking into account the fixed factors of season (winter, spring, summer, and autumn) and different temperatures in each season, and the correlation between both factors. 

## Results

### Oxygen fluxes

**Fig. 4:**
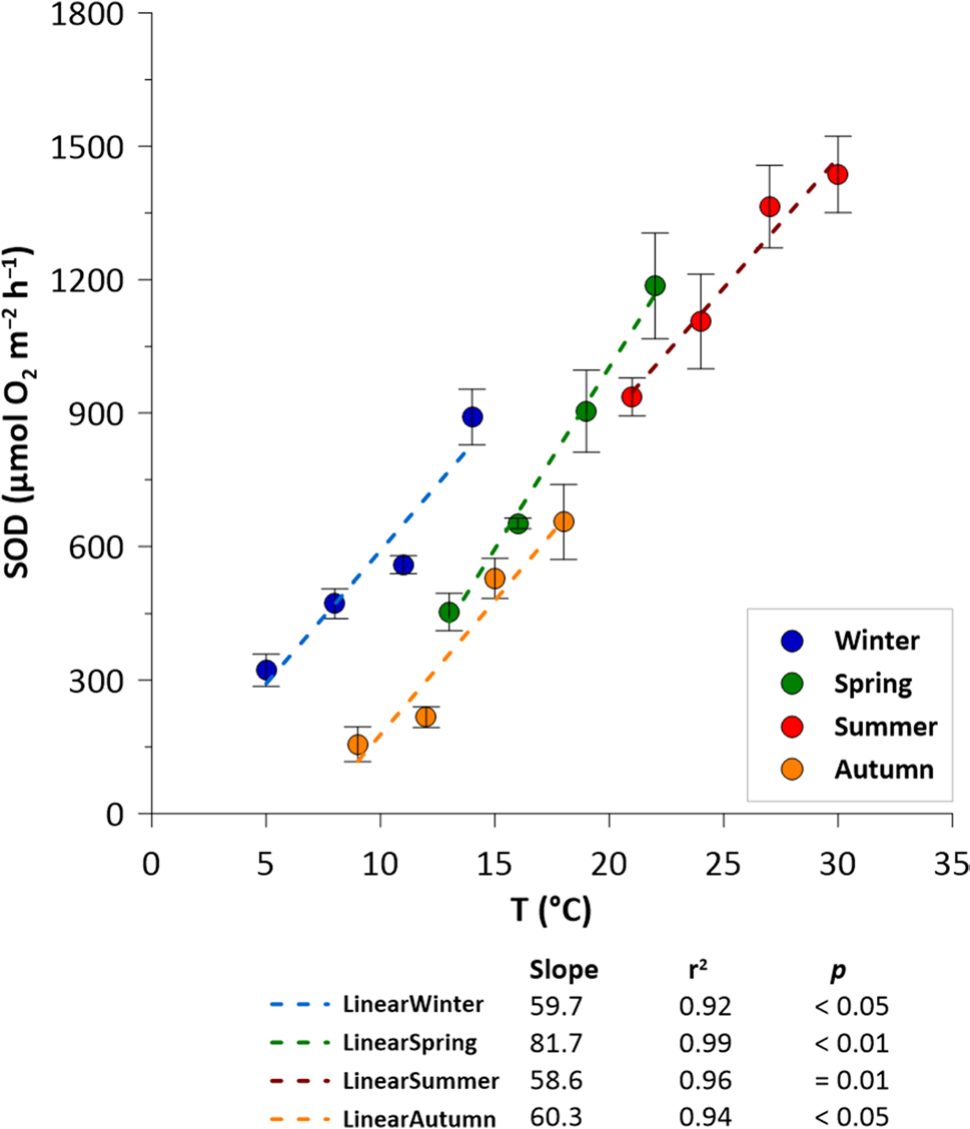
Sediment oxygen demand (SOD, μmol O_2_ m^−2^ h^−1^) measured along the temperature gradients in the four seasons. Average values ± standard deviations are reported (n = 5). Trend slopes, coefficient of determination (*r*^2^), and *p-value* of linear regression models are reported at the bottom of the figure

The oxygen concentration measured *in situ* during the four sampling campaigns ranged from 8.3 mg L^−1^ in summer, to 11.7 mg L^−1^ in winter, corresponding to approximately 100% saturation of the water column throughout the year (Table 2). The sediment oxygen demand increased with temperature in all seasonal incubations (Fig. 4). The average values in winter, spring, summer, and autumn were 561 ± 120, 799 ± 159, 1210 ± 116, and 389 ± 120 μmol O_2_ m^−2^ h^−1^, respectively. The raise along the temperature gradient averaged at 60 μmol O_2_ m^−2^ h^−1^ °C^−1^ in all seasons, except in spring when the highest SOD values were detected. The SOD in spring ranged from 453 ± 42 to 1186 ± 119 μmol O_2_ m^−2^ h^−1^ at 13 and 22 °C, respectively, and increased along the temperature gradient with a raise of 82 μmol O_2_ m^−2^ h^−1^ per degree. In summer incubation, SOD had the highest values due to the highest temperatures, from 936 ± 43 μmol O_2_ m^−2^ h^−1^ at 21 °C to 1937 ± 86 μmol O_2_ m^−2^ h^−1^ at 30 °C. On the contrary, the lowest values appeared during the autumn incubation, when SOD fluxes ranged between 156 ± 39 and 955 ± 84 μmol O_2_ m^−2^ h^−1^ (at 9 and 18 °C, respectively). Finally, the winter SOD ranged from 323 ± 36 to 891 ± 62 μmol O_2_ m^−2^ h^−1^ at 5 and 14 °C, respectively.

**Table 2:** Chemical conditions (temperature, O_2_, and N nutrient concentrations) at Pontelagoscuro site during the four seasonal campaigns. Standard deviations are reported (±)

Seasons	Sampling day	Temperature (°C)	O_2_ (mg L^−1^)	NO_3_^−^ (μM)	NO_2_^−^ (μM)	NH_4_^+^ (μM)
*Winter*	15th February	8	11.7	201 ± 0.5	2 ± 0.1	4 ± 1
*Spring*	3rd May	20	10.9	126 ± 6	1 ± 0.1	5 ± 1
*Summer*	19th July	29	8.3	40 ± 3	1 ± 0.3	4 ± 1
*Autumn*	14th November	14	10.2	190 ± 4	2 ± 0.5	< 0.5

The results of the spring experiments were within the range of two extreme seasons, i.e. summer and winter. In fact, the SOD at the highest spring temperature had the same values as those of the summer SOD at the lowest temperature, and the lowest spring temperature had the same values as the highest winter temperature. The lowest values, 156 ± 39 and 216 ± 23 μmol O_2_ m^−2^ h^−1^, were recorded during the autumn period at the first two temperatures tested, 9 and 12 °C, respectively, due to the low experimental temperature and initial O_2_ concentration in situ (Table 2).

### Denitrification and DNRA rates

**Fig. 5:**
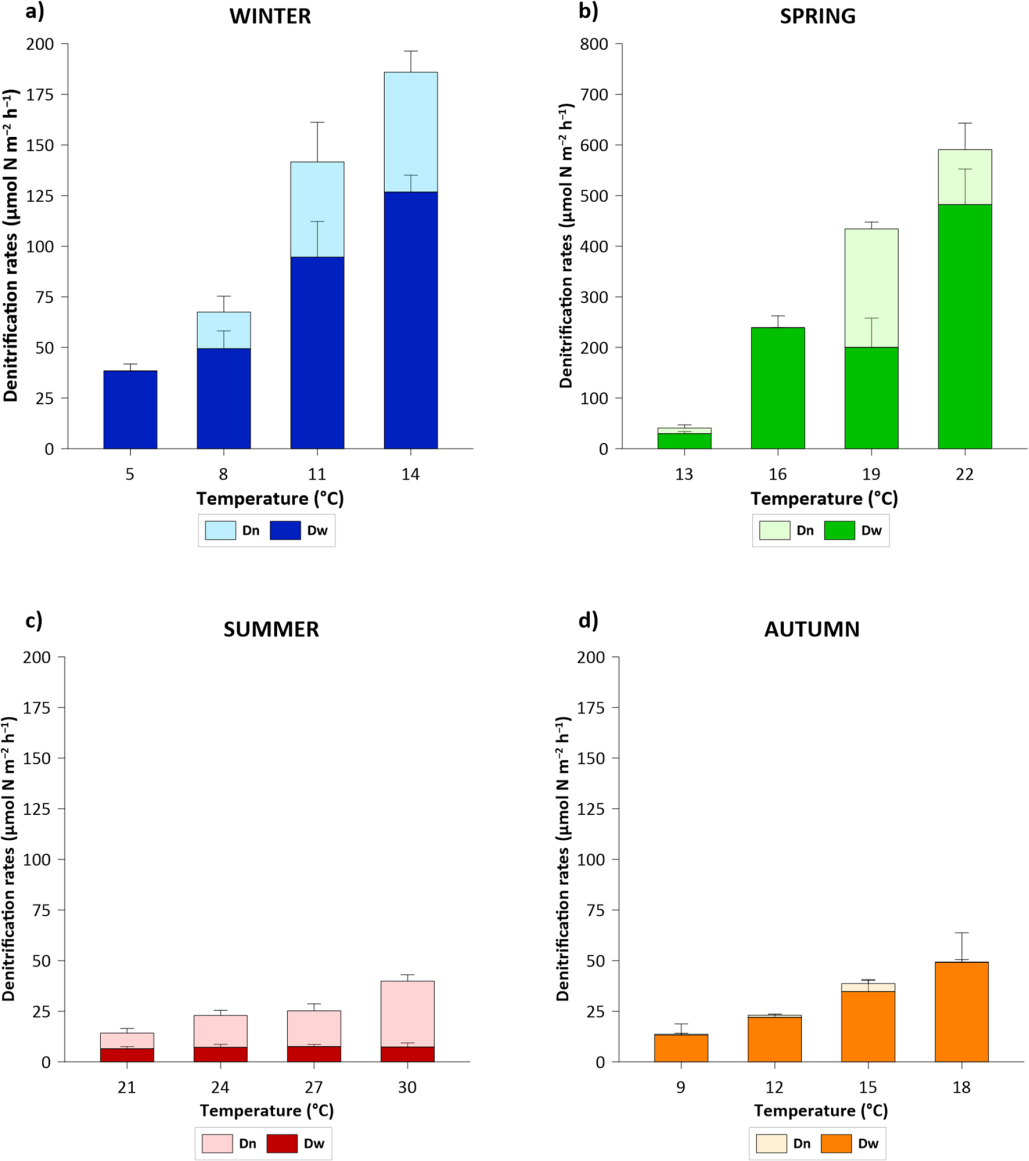
Total denitrification rates (μmol N m^−2^ h^−1^) splitted into Dw and Dn measured along the temperature gradients in the four seasons: **a** winter; **b** spring, **c** summer; **d** autumn (note the different scale of the y-axis). Average values ± standard deviations are reported (n = 5). Dw = denitrification rate of NO_3_^−^ diffusing from the water column to the anoxic sediment layer. Dn = denitrification rate coupled with nitrification

Denitrification and DNRA rates showed wide seasonal variations related to both NO_3_^−^ availability and temperature. Total denitrification rates increased along the experimental temperature gradient that was set in each season (Fig. 5). In winter, the total denitrification rates ranged from 38 ± 3 μmol N m^−2^ h^−1^ at 5 °C to 186 ± 16 μmol N m^−2^ h^−1^ at 14 °C, with a rise of 16 μmol N m^−2^ h^−1^ °C^−1^. The highest rates were measured in spring incubation, increasing from 41 ± 5 to 591 ± 29 μmol N m^−2^ h^−1^ at 13 and 22 °C, respectively, with a rise of 61 μmol N m^−2^ h^−1^ °C^−1^. The summer rates increased from 14 ± 8 μmol N m^−2^ h^−1^ at 21 °C to 40 ± 5 μmol N m^−2^ h^−1^ at 30 °C, with a slight increase, i.e., only 3 μmol N m^−2^ h^−1^ °C^−1^ and, finally, the autumn denitrification rates ranged from 14 ± 5 to 49 ± 14 μmol N m^−2^ h^−1^ at 9 and 18 °C, respectively, with a raise of 4 μmol N m^−2^ h^−1^ °C^−1^. Dw dominated Dn, accounting for an average of 76% of Dtot in the winter and spring incubations. In fact, in winter Dw ranged from 38 ± 3 to 127 ± 8 μmol N m^−2^ h^−1^ at 5 and 14 °C, respectively, while it showed the highest values in spring incubations, ranging from 30 ± 4 to 482 ± 70 μmol N m^−2^ h^−1^ at 13 and 22 °C, respectively. In contrast, in the summer incubations, the Dw (denitrification of NO_3_^−^ diffusing from the water column to the sediments) was systematically lower than Dn (denitrification coupled to nitrification), representing an average of 31% of Dtot. During the summer experiment, Dn increased along gradient temperature, ranging from 8 ± 3 to 32 ± 3 μmol N m^−2^ h^−1^ at 21 and 30 °C, respectively, while the same upward trend was not detected for Dw. Autumn Dw represented 95% of Dtot and increased along the temperature gradient, ranging from 13 ± 5 to 49 ± 15 μmol N m^−2^ h^−1^ at 9 and 18 °C, respectively. A strong denitrification response to temperature was observed, with an increase in the temperature gradient in all seasons (*p* < 0.001; Table 3). Statistical analysis showed a strong correlation between the N process and temperature in each season (Table 3), and the interaction between temperature and season was also significant, ; nevertheless, the average rates differed among seasons (Table 3), due to the different seasonal NO_3_^−^ availability.

**Table 3:** Effects of factors temperature, season and their interaction on SOD, denitrification (Dw, Dn, and Dtot), and DNRA rates (DNRAw, DNRAn, and DNRAtot) based on LME models. Significant *p*-values (<0.05) are bolded

Variable	Temperature		Season		Temperature × Season	
	F	*p*	F	*p*	F	*p*
SOD	548.8	**<0.0001**	24.9	**<0.0001**	1.3	0.2835
Dw	3.1	0.0809	79.3	**<0.0001**	26.5	**<0.0001**
Dn	5.1	**0.0264**	14.7	**<0.0001**	5.6	**0.0018**
Dtot	40.5	**<0.0001**	434.9	**<0.0001**	149.9	**<0.0001**
DNRAw	5.7	**0.0194**	12.9	**<0.0001**	0.7	0.5429
DNRAn	5.9	**0.0180**	8.7	**0.0001**	2.4	0.0729
DNRAtot	0.0	0.9627	18.0	**<0.0001**	2.3	0.0843

**Fig. 6:**
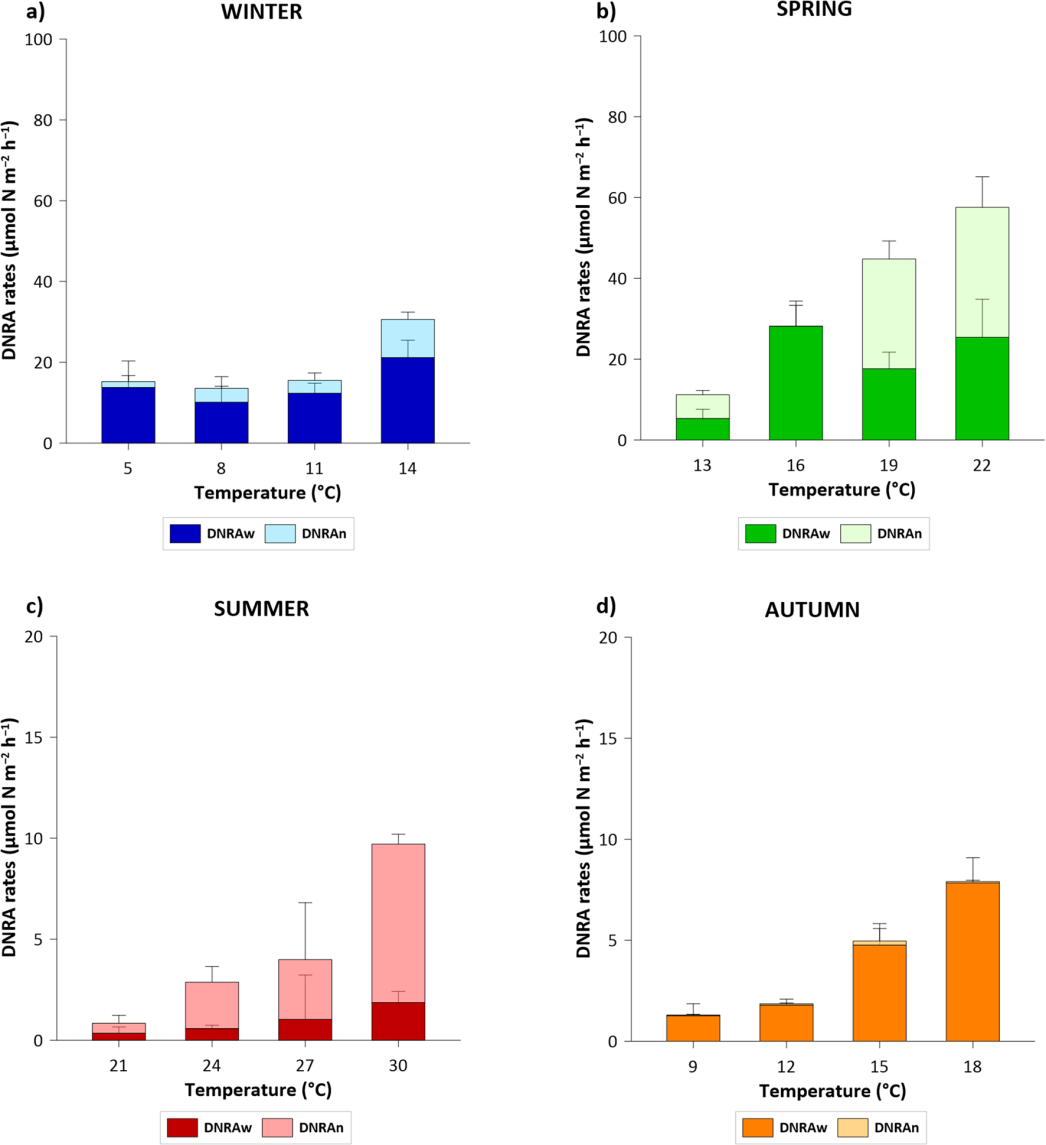
Total dissimilatory nitrate reduction to ammonium (DNRA) rates (μmol N m^−2^ h^−1^) splitted into DNRAw e DNRAn, measured along the temperature gradients in the four seasons: **a** winter; **b** spring, **c** summer; **d** autumn (note the different scale of the y-axis). Average values ± standard deviations are reported. DNRAw = the direct DNRA of NO_3_^−^ from the water column. DNRAn = the DNRA rate coupled with nitrification

On average, the denitrification rates were one order of magnitude higher than the DNRA rates. DNRA increased along the temperature gradient in all seasons except winter, when the rates remained constant at the first three temperatures of the series (average 14 μmol N m^−2^ h^−1^), increasing up to 31 ± 6 μmol N m^−2^ h^−1^ at the last one (14 °C). The highest DNRA rates were measured in spring, ranging from 11 ± 3 to 53 ± 24 μmol N m^−2^ h^−1^ at 13 and 22 °C, respectively. The rates in summer and autumn were very low in comparison to the other seasons (Fig. 6; note the difference in scale of the y-axis) and follow a significant linear increase with temperature, from < 2 to 10 ± 1 μmol N m^−2^ h^−1^, at 21 and 30 °C, in summer, and from  < 2 to 8 ± 1 μmol N m^−2^ h^−1^, at 9 and 18 °C, in autumn, respectively.

In winter and spring, DNRAw increased along the temperature gradient (Fig. 6) and represented on average 77 and 64% of DNRAtot, respectively. In summer, the DNRA rates of NO_3_^−^ diffusing from the water column to the anoxic sediment layer (DNRAw) increased slightly along the temperature gradient but represented less than 30% of DNRAtot. Finally, in autumn, DNRAw increased along the temperature gradient and accounted for >95% of the DNRAtot.

## Discussion and conclusion

An increase in the water temperature of a river has several consequences. As water temperature increases, oxygen solubility and diffusion into the sediment decrease (Butcher and Covington 1995; Rajesh and Rehana 2022; Veraart et al. 2011; Muruganandam et al. 2023). Simultaneously, temperature warming stimulates sediment respiration rates and SOD, which in turn results in the vertical extension of the hypoxic-anoxic area within superficial sediments. Thus, this thicker anoxic sediment layer becomes suitable for denitrification because enzymes that sequentially reduce NO_3_^−^ to N_2_, i.e., NO_3_^−^, NO_2_^−^, NO, and N_2_O reductase (Bonnett et al. 2013; Hobbs et al. 2013; Adouani et al. 2015).

In this study, denitrification responded positively to water warming, increasing along a temperature gradient in each season. There was a significant interaction between temperature and season (Table 3), indicating that the effect of temperature on denitrification was also dependent on other seasonal factors such as NO_3_^−^ availability (Myrstener et al. 2016), which influenced the partitioning of total denitrification into Dw and Dn. On average, increasing temperature and higher NO_3_^−^ availability in the water column stimulate anaerobic processes, allowing the contribution of Dw to increase with respect to Dn (Dong et al. 2000). In fact, it has been widely reported that total denitrification rates are mainly supported by Dw when NO_3_^−^ concentrations in the water column exceed 50 μM (Piña-Ochoa and Álvarez-Cobelas 2006; Nizzoli et al. 2010; Racchetti et al. 2011), according to the winter and spring results. In winter, the total denitrification rates and, consequently, the Dw rates, were low owing to low temperatures, despite the high NO_3_^−^ concentrations compared with the other seasons. In spring, the total denitrification and Dw rates were the highest due to NO_3_^−^ availability, increasing along the temperature gradient. In contrast, summer denitrification rates were limited by NO_3_^−^ availability, despite high temperatures. The exceptionally low flows that characterized the Po River in the summer of 2022 (Montanari et al. 2023) led to a reduction in nutrient runoff from the basin (Cozzi et al. 2018; Viaroli et al. 2018) and consequently limited NO_3_^−^ availability in water, resulting in a greater relevance of Dn compared to Dw, as previously reported also in the Po delta course, where Dn accounted for >50% of Dtot (Gervasio et al. 2023).

Another exception occurred in autumn, when denitrification rates were low despite the water temperature and NO_3_^−^ availability (Tables 1, 2). However, although no specific measurements were made in this study, our results support the hypothesis that the sediment content of labile organic matter was lower in autumn (Table 4) than in other seasons, as a result of a decrease in river primary productivity and sedimentation of labile phytoplanktonic material and an increase in lignocellulosic debris transport from the catchment after moderate rainfall, in the week prior to sampling. It is hypothesized that the shift in the ratio of highly biodegradable autochthonous material to refractory lignocellulosic material from the catchment has slowed heterotrophic bacterial metabolism in the sediment and hence NO_3_^−^ reduction processes (Hu et al. 2019; Warneke et al. 2011). This hypothesis is supported by the low SOD flux measured in autumn (Fig. 4). SOD is strongly related with the availability of labile organic matter in river sediments, the mineralization of which primarily involves oxygen (Hargrave 1972). Thus, oxygen consumption can be considered a proxy for mineralization rates (Seiter et al. 2005; Song et al. 2016). The low SOD in autumn at the same temperatures and with approximately the same sediment total organic matter content measured in spring, when the SOD was almost three times higher, highlights the role of organic matter quality (Myrstener et al. 2016) and variation throughout the year in the Po River.

**Table 4:** Physical characteristics (organic matter, volumetric water content, density, and porosity) of the Po River sediments in each season. Standard deviations are reported (±)

Seasons	Sampling day	OM (%)	VWC (%)	Density (g mL^−1^)	Porosity
*Winter*	15th February	1.6 ± 0.5	37 (±) 14	1.6 (±) 0.2	0.6 (±) 0.1
*Spring*	3rd May	0.6 ± 0.01	23 (±) 2	1.9 (±) 0.1	0.4 (±) 0.04
*Summer*	19th July	0.4 ± 0.03	18 (±) 1	2.0 (±) 0.1	0.4 (±) 0.03
*Autumn*	14th November	1.7 ± 0.4	33 (±) 2	1.8 (±) 0.2	0.6 (±) 0.1

The denitrification rates measured in this study overlap with those of other large rivers worldwide, although the variability found in the literature studies may be due not only to variable environmental conditions but also to different experimental approaches resulting in rates varying over several orders of magnitude (Piña-Ochoa and Álvarez-Cobelas 2006; Reisinger et al. 2016; Qi and Liu 2023). As recently reviewed by Gervasio et al. (2022), in a global hotspot of NO_3_^−^ pollution such as the Po River basin, denitrification has been extensively measured in several types of aquatic ecosystems (e.g., wetlands, canals, lagoons, rivers, and lakes) and its regulating factors have been diffusely studied. The range of denitrification rates measured in the sediments of the Po River overlapped with estimates in aquatic environments heavily influenced by the surrounding agricultural landscapes (connected wetlands and rivers), resulting in an increased availability of NO_3_^−^ and organic carbon, but higher than those found in isolated wetlands, lakes, and coastal lagoons.

Similar to denitrification, DNRA rates were temperature dependent in each seasonal experiment. The total DNRA rates increased along the temperature gradient in each season (Roberts et al. 2014), with the highest rates measured in spring, followed by winter, autumn, and summer DNRA rates. The two contributions of DNRA, i.e. DNRAw and DNRAn, followed the same increase along the temperature gradient, with the exception of DNRAw in summer, due to lower NO_3_^−^ availability in the water column. In each season, DNRAw dominated DNRAn, except in summer, when the low water NO_3_^-^ availability due to the extremely dry summer of 2022 resulted in higher DNRAn rates compared to DNRAw. A previous study showed that DNRA exceeded denitrification in organic-rich brackish sediments of the Po River (Gervasio et al. 2023), with rates positively related to the C/N ratio, as a consequence of stimulation by labile carbon availability (Nizzoli et al. 2010). The sediments of the Po River main course, at the section sampled in this study, were poor in organic matter in all seasons (Table 1), particularly the minimum values were measured in summer, highlighting the conditions under which DNRA is not favored (Wei et al. 2020). These results contradict those of other studies showing that increasing temperatures can promote reducing conditions in the sediment by favoring DNRA over denitrification (Yin et al. 2002). In winter, the DNRA had similar values at three of the four experimental temperatures. This was attributed to the high oxygen concentration in the river water column, which is generally well mixed and well-oxygenated, especially in winter (Table 2) (Frascari et al. 2006). High oxygen availability, combined with low sediment organic carbon and low SOD, is such as to result in the complete oxidation of the sandy sediments, limiting the activity of DNRA bacteria to micro-niches and resulting in low rates, that were unaffected by the experimental temperature increase (Kraft et al. 2014). Finally, in autumn, the DNRA rates were low despite NO_3_^−^ availability (Table 2). This evidence is consistent with the hypothesis previously discussed, regarding the importance of the labile organic matter fraction of the total organic matter in river sediments for DNRA and denitrification (Jiang et al. 2020; Guo et al. 2022; Jaiswal et al. 2023).

Overall, denitrification was found to be the main process responsible for the removal of NO_3_^−^ from the Po River with a positive trigger from temperature increase, especially in spring when NO_3_^−^ availability is maximal, as well as the risk of eutrophication in the Adriatic Sea. Instead, the temperature increase did not favor N recycling via DNRA to the extent that it exceeded denitrification, which generally occurs in sediments under strongly reducing conditions (Yuan et al. 2023). In the Po sediments, DNRA contributed on average of 13% to the total NO_3_^−^ dissimilatory reduction, while most of the NO_3_^−^ was permanently removed by denitrification, especially in autumn, when denitrification accounted for >90% of the total NO_3_^−^ removal.

In conclusion, the direct link between water warming induced by climate change and positive denitrification response, described in this study, may have implications for water quality improvement in the Adriatic Sea, due to the potential reduction of N loads especially in spring, when riverine nutrients trigger the most eutrophication. The present outcomes suggest that recent increases in Po River water temperature may have increased the rate of NO_3_^−^ loss via denitrification in the lowland reach sediments and ultimately caused the downward trends of N loads discharged to the Adriatic Sea, observed in recent decades (Gervasio et al. 2022; Soana et al. 2023). Future studies should seek direct confirmation of this conclusion by extending to the ecosystem scale the experimental rates measured along water temperature gradients in sections characterized by different substrate availability (i.e., NO_3_^−^ in the water column and labile organic matter in the sediments). Their multiple functional relationships make in fact the effects of climate change on rivers very complex to analyze and difficult to predict. Further research is needed to examine how other climatic factors, such as reduced discharge and extreme rainfall events, may interact with temperature increases in terms of both the dynamics of nutrient load generation and nutrient export to the sea from temperate and Mediterranean basins. This is a key issue for the effective implementation of environmental policies to control eutrophication and protect coastal zones.
